# Patient‐reported outcome measures (PROMs): A review of generic and condition‐specific measures and a discussion of trends and issues

**DOI:** 10.1111/hex.13254

**Published:** 2021-05-05

**Authors:** Kate Churruca, Chiara Pomare, Louise A. Ellis, Janet C. Long, Suzanna B. Henderson, Lisa E. D. Murphy, Christopher J. Leahy, Jeffrey Braithwaite

**Affiliations:** ^1^ Centre for Healthcare Resilience and Implementation Science Australian Institute of Health Innovation Macquarie University Sydney NSW Australia; ^2^ The Australian Commission on Safety and Quality in Health Care Sydney NSW Australia

**Keywords:** patient safety, patient‐reported outcome measure, PROM, review

## Abstract

**Background:**

Patient‐reported outcome measures (PROMs) are questionnaires that collect health outcomes directly from the people who experience them. This review critically synthesizes information on generic and selected condition‐specific PROMs to describe trends and contemporary issues regarding their development, validation and application.

**Methods:**

We reviewed academic and grey literature on validated PROMs by searching databases, prominent websites, Google Scholar and Google Search. The identification of condition‐specific PROMs was limited to common conditions and those with a high burden of disease (eg cancers, cardiovascular disorders). Trends and contemporary issues in the development, validation and application of PROMs were critically evaluated.

**Results:**

The search yielded 315 generic and condition‐specific PROMs. The largest numbers of measures were identified for generic PROMs, musculoskeletal conditions and cancers. The earliest published PROMs were in mental health‐related conditions. The number of PROMs grew substantially between 1980s and 2000s but slowed more recently. The number of publications discussing PROMs continues to increase. Issues identified include the use of computer‐adaptive testing and increasing concerns about the appropriateness of using PROMs developed and validated for specific purposes (eg research) for other reasons (eg clinical decision making).

**Conclusions:**

The term PROM is a relatively new designation for a range of measures that have existed since at least the 1960s. Although literature on PROMs continues to expand, challenges remain in selecting reliable and valid tools that are fit‐for‐purpose from the many existing instruments.

**Patient or public contribution:**

Consumers were not directly involved in this review; however, its outcome will be used in programmes that engage and partner with consumers.

## 1 INTRODUCTION

Over the last few decades, health‐care systems have increasingly recognized patients' perspectives as fundamental to ensuring that services are of a high quality and delivered in an equitable and safe way.^1^ The expanding use of patient‐reported outcome measures (PROMs) has been part of this shift.^2^, ^3^ PROMs are standardized questionnaires that collect information on health outcomes directly from patients, including about symptoms, health‐related quality of life and functional status. In addition to standardization, PROMs should ideally undergo psychometric validation to ensure that they accurately reflect the outcomes they purport to measure and that they can reliably assess changes over time.^4^

PROMs were originally developed for use in research, particularly clinical trials assessing the effectiveness of treatments.^5^ Over time, their applications have broadened to include the following: supporting clinical decision making, prioritizing patients for surgical procedures, comparing outcomes among health‐care providers, stimulating quality improvement and evaluating practices and policies.^2^, ^3^, ^6^, ^7^, ^8^ Evidence that the routine use of PROMs, at least in an oncological setting, leads to better outcomes for patients is inconclusive, but they do appear to improve patient‐provider communication and patient satisfaction.^6^, ^9^ Potential benefits of these measures rely on them being rigorously developed, relevant to patients and well‐validated.^10^

Broadly, PROMs fall into two main categories: condition‐specific and generic. The latter measures health concepts that are relevant to a wide range of patient groups, enabling aggregation and comparisons across varied conditions and settings. An example is the EQ‐5D; developed by the EuroQol Group,^11^ it includes five questions asking after the patient's health that day, mobility, self‐care, usual activities, pain/discomfort and anxiety/depression. Condition‐specific PROMs capture elements of health relevant to a particular patient group or condition. For example, the European Organisation for Research and Treatment of Cancer^12^ developed a widely used PROM that assesses the quality of life of patients with cancer (EORTC QLQ‐30). There is a core generic questionnaire for all patients with cancer, as well as modules targeting symptoms and outcomes of different cancer diagnoses (eg the European Organization for Research and Treatment of Cancer Quality of Life Questionnaire Lung Cancer Module (EORTC QLQ‐LC13)^13^).

Condition‐specific and generic PROMs are both important for understanding and improving patient care at multiple levels of the health‐care systems. Past literature reviews on PROMs have focused on identifying measures related to specific conditions (eg epilepsy^14^) or populations (eg children and adolescents^15^). A review that critically synthesizes information on PROMs and considers trends and issues in this literature has thus far been lacking. This paper aims to address this gap by evaluating trends in PROMs and their publication and discusses contemporary issues that relate to the development, validation and application in health care.

## 2 METHODS

We used a rapid review methodology to synthesize the evidence on generic and condition‐specific PROMs, identifying measures and evaluating trends and issues. Rapid review is a form of evidence synthesis that streamlines traditional systematic review methods in a shortened time frame.^16^ The approach was selected due to the fast‐moving nature of the field and the breadth of focus of this review. The Preferred Reporting Items for Systematic Reviews and Meta‐analyses (PRISMA) guidelines were used to guide the methodological design.^17^ A critical review approach^18^ was adopted to evaluate trends and contemporary issues in the use of PROMs. This rapid review was conducted for a lead safety and quality organization in Australia, the Australian Commission on Safety and Quality in Health Care. Although consumers were not directly involved in this review, the Commission engages and partners with consumers to deliver its work programmes. This review is part of its work to support the use of PROMs to ‘drive quality improvement in a way that brings patients’ voices and outcomes to the fore’.^19^

### 2.1 Search strategy

Following consultation with a research librarian and an examination of past systematic reviews on PROMs,^15^, ^20^ keywords and MeSH terms were developed. The search of academic literature was limited to papers published in English and published over the last 30 years (1989‐2019). This period coincides with the rapid growth in the use of PROMs.^21^ However, PROMs originally developed prior to the 30‐year search window were included where they were reported on in papers published during this period. The search was also restricted to journal articles (ie conference papers and books were excluded). The search was run on four databases (MEDLINE, EMBASE, PsycINFO, CINAHL) in conjunction with a search of the grey literature and snowballing of reference lists and past reviews. An example of the search strategy used is displayed in Table 1. The search was carried out on 15 May 2019.

**TABLE 1:** Keywords and MESH terms

Database	Keywords and MeSH
MEDLINE	“patient?reported outcome” OR “PROM” OR MeSH term “patient reported outcome measures” AND “psychometr*” OR “reliability” OR “valid*” AND “questionnaire” OR “tool” OR “scale” OR “survey” OR “measure” OR “instrument” OR “interview”

### 2.2 Eligibility criteria

PROMs were included in the review if they met the following criteria: (1) standardized instrument/survey that is used for measuring health outcomes (eg symptoms, quality‐of‐life, functional status) reported directly by the patient and using a range of modes of delivery, including computerized‐adaptive testing (CAT); (2) validated, that is, there was published statistical analyses establishing reliability (eg Cronbach's alpha^22^) and validity of the scale(s), including construct validity (eg factor analyses, item‐response modelling, convergent and discriminant validity), criterion‐related validity (eg concurrent and predictive validity), or analyses of known group differences; (3) validation analyses were conducted on an English language version of the instrument, either in the original validation paper or subsequently and (4) the measure assesses generic health status OR is a condition‐specific PROM from one of the included conditions (see Results, Table 2). To be included, it was not required that a survey be described as a ‘PROM’ in its original development, but it needed to measure patient‐reported health outcomes as per criterion (1).

**TABLE 2:** Included PROMs by disease group

Disease group	Diseases	PROMs
Musculoskeletal	Back pain and problems, Osteoarthritis, Rheumatoid arthritis	42
Generic		39
Cancer	Lung cancer, Bowel cancer, Breast cancer, Prostate cancer, Pancreatic cancer, Brain and central nervous system cancer	32
Gastrointestinal	Chronic liver disease, Inflammatory bowel disease (IBD)	30
Neurological	Dementia, Epilepsy, Migraine, Parkinson's disease, Delirium	25
Mental health	Anxiety, Depression, Psychological distress, Alcohol use disorders	23
Cardiovascular	Coronary heart disease, Stroke, Atrial fibrillation and flutter, Non‐rheumatic valvular disease, Cardiomyopathy, Hypertension	22
Reproductive and maternal	Genital prolapse, Polycystic ovarian syndrome, Heavy menstrual bleeding	20
Hearing and vision	Hearing loss, Vision loss, Cataracts	15
Respiratory	Chronic obstructive pulmonary disease, Asthma	14
Endocrine	Diabetes	13
Injuries and trauma	Burns	10
Infectious	HIV/AIDs	10
Kidney and urinary	Chronic kidney disease	7
Skin	Dermatitis and eczema	7
Oral	Dental caries, Severe tooth loss	4
Blood and metabolic	Iron‐deficiency anaemia	2
Total		315

For criterion (4), conditions were selected by examining the Australian Institute of Health and Welfare's (AIHW’s) list of high burden diseases,^23^ which largely reflected international trends.^24^ Reproductive and maternal conditions were also added based on consultation with the project funder. These conditions are grouped by International Classification of Diseases (ICD‐10) disease groups.^25^

### 2.3 Additional searches: Snowballing, scoping published reviews and grey literature

When reviewing validation papers, researchers noted any additional potential PROMs, adding them to the list for scoping (ie snowballing). Searches for other PROMs in Scopus, Google Scholar and Google Search were also performed using terms for both specific conditions and broader disease groups. Websites that list PROMs, such as the International Consortium for Health Outcomes Measurement (ICHOM),^26^ were checked. Researchers also searched for recent systematic and other reviews on the topic, reading these papers to ensure as far as possible no relevant PROMs were missed.

### 2.4 Screening and data extraction

Citations returned from the database searches were downloaded into Endnote and duplicates were removed. Titles and abstracts were then exported to Microsoft Excel for a simultaneous process of screening against inclusion criteria and preliminary data extraction. Preliminary data extraction involved documenting the relevant disease group (where applicable, from Table 2) and condition(s) reported in the abstract, plus any PROMs mentioned by name, for all citations meeting the inclusion criteria. In the few cases where there was not sufficient information in the abstract to assess eligibility or extract data, the full text of the paper was examined.

In the second stage, PROMs labelled with conditions from our list were reviewed in detail. These were distributed amongst members of the research team by disease group (eg KC reviewed all PROMs for the included musculoskeletal conditions). Researchers searched for the original validation paper(s) for each PROM to fill in data on country, year and process of validation and PROM domains/dimensions; they documented the number of times the validation paper had been cited in Google Scholar as an indicator of how widely the PROM was used in published research. Researchers then scoped more recent papers using the PROM, selecting an exemplar study, ideally from 2012 or later to indicate currency. Reports or other clinical or policy‐focused evidence of use were also searched for online and documented when identified. While conducting the review and extracting data, researchers recorded trends and issues in the development, validation and application of PROM. These included: recurrent patterns in the types of PROMs published (generic and condition‐specific), the terminology used to describe them, modes of delivery and contemporary issues in their use (eg validity, use of technology). These were identified from reviewing the PROMs papers and examining the systematic reviews used for snowballing.

## 3 RESULTS

**FIGURE 1:**
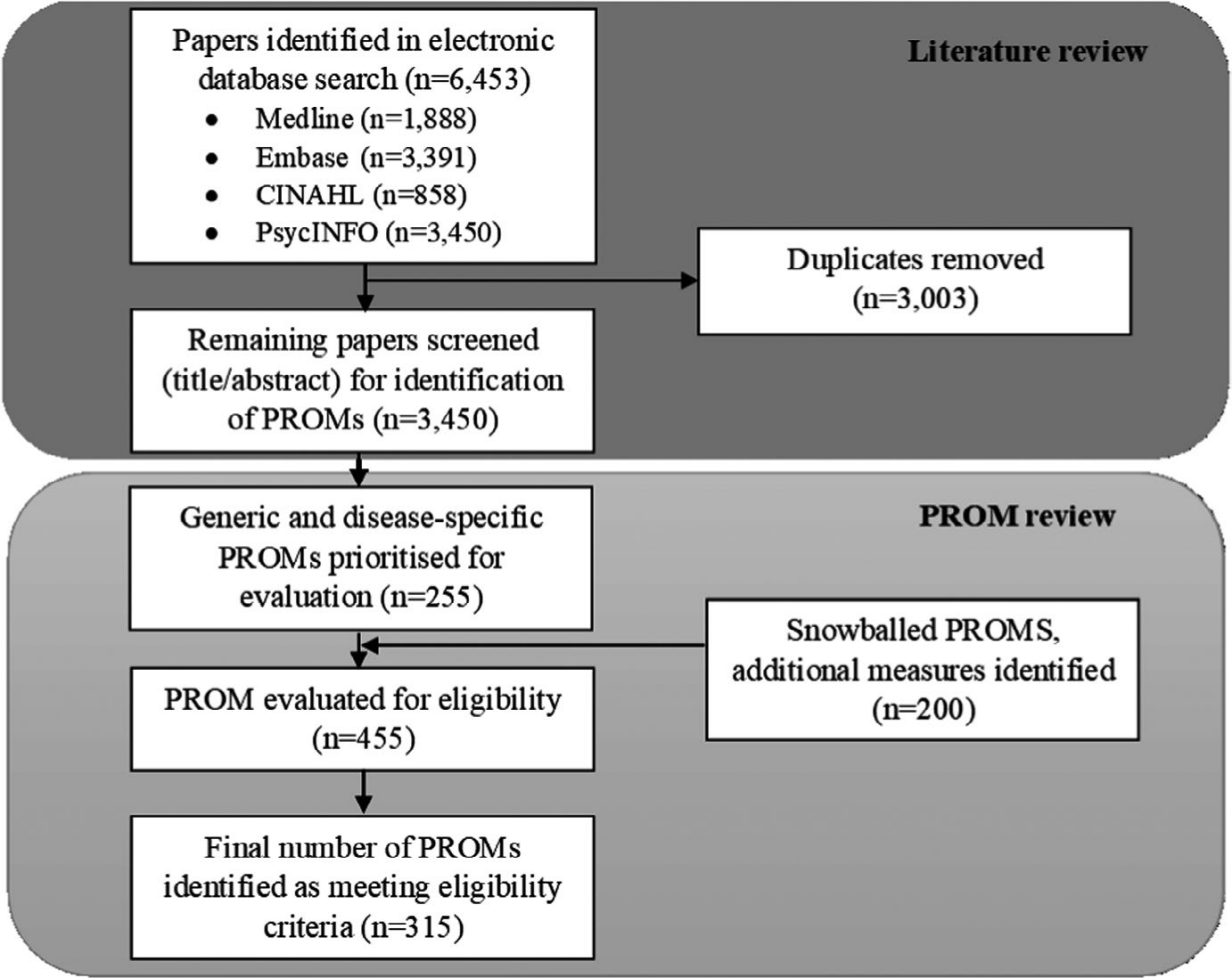
Flow chart of the rapid review to identify validated PROMs

A total of 6453 citations were returned from the database search. Following removal of duplicates, 3450 titles/abstracts were exported to Excel for screening and preliminary data extraction (Figure 1). This led to the identification of 255 PROMs, both generic and from conditions in Table 2.

A further 200 PROMs were identified through additional searches, amounting to 455 PROMs evaluated. Of these, 315 met all inclusion criteria and were included in this review (Appendix S1). PROMs were excluded at this stage for a number of reasons including insufficient evidence of validation or lack of validation in English, measures were a clinical assessment tool (ie used by clinicians) or not yet sufficiently validated for use as a PROM, the measure was too generally described or lacked appropriate standardization to be adequately validated (eg general reports of numeric rating scales, visual analogue scales, the Patient Global Assessment^27^), or the information available online was too limited to assess eligibility.

The number of PROMs included in the review by disease group is shown in Table 2. As can be seen, the highest number of PROMs was identified for musculoskeletal conditions and for generic PROMs, followed by cancer and gastrointestinal conditions. Only two PROMs were identified covering iron‐deficiency anaemia, the only blood and metabolic condition included in the condition list.

**FIGURE 2:**
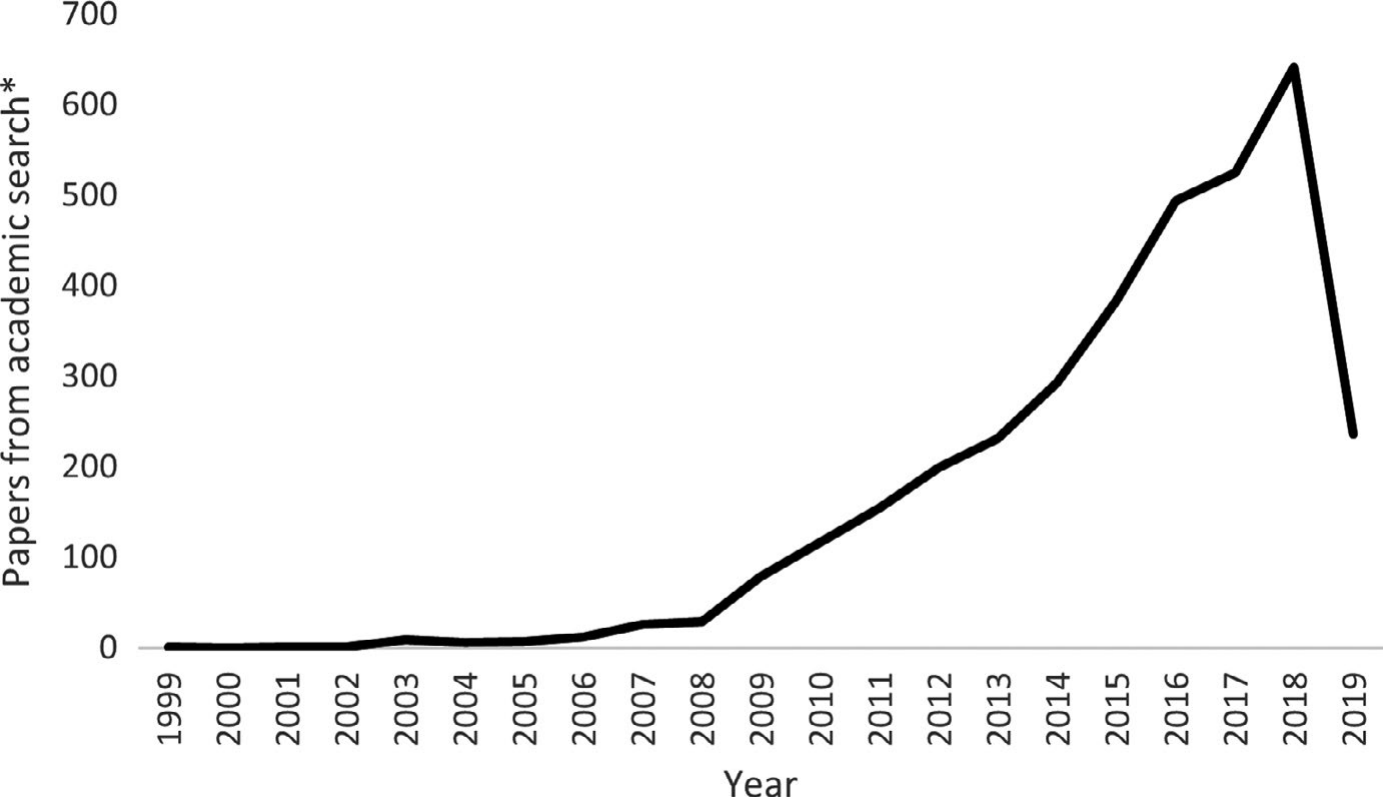
Papers about PROMs over time. *Number of papers from academic literature search after duplicate removal (n = 3450), which used the terms ‘PROM’ or ‘patient‐reported outcome’. 2019 is an incomplete year

**FIGURE 3:**
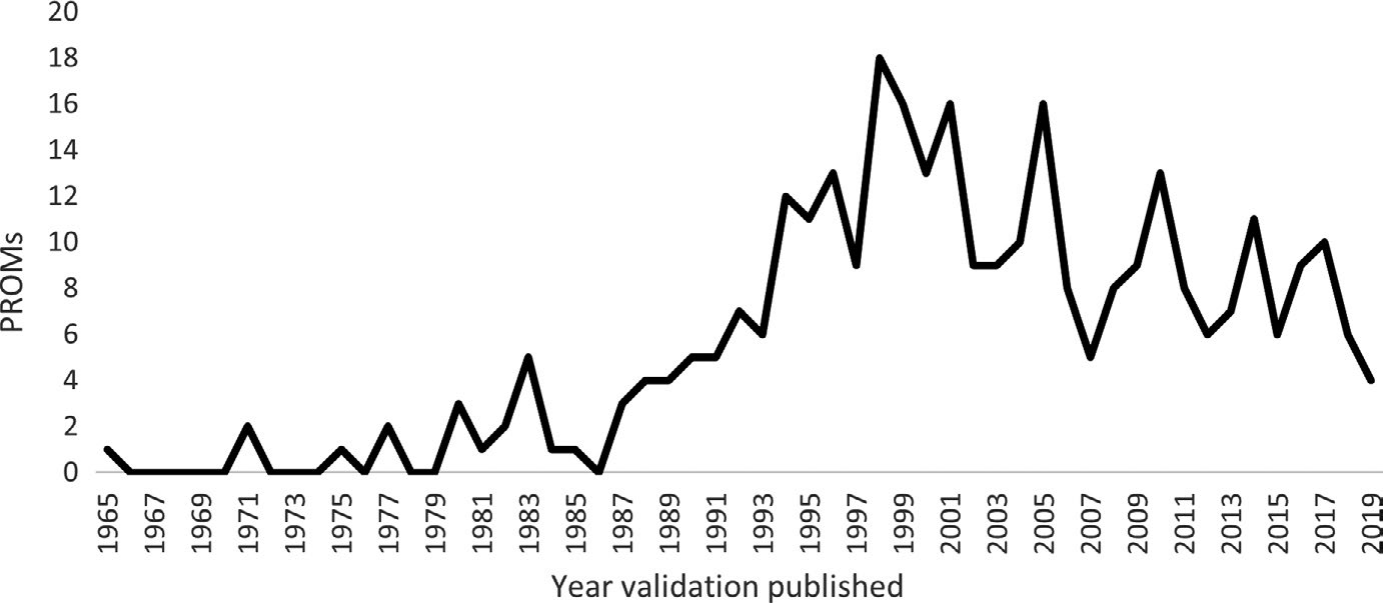
Publication of validation papers for included PROMs over time. PROMs from prior to 1999 were identified only by grey literature search or being cited in secondary academic sources. 2019 is an incomplete year

In compiling PROMs, trends over time were examined among measures and publications. Despite the date range of 1989‐2019, no papers came up from the academic literature search prior to 1999, indicating the time dependent nature of the term ‘patient‐reported outcome measure’. However, after this time publications using this terminology increased rapidly (see Figure 2). From reviewing information in these papers, as well as searches of grey literature, we were able to identify validated PROMs from a much wider date range, with the first validation paper for a PROM included in this review being from 1965. The number of validated PROMs being published increased until the 2000s, when this trend plateaued (see Figure 3).

**FIGURE 4:**
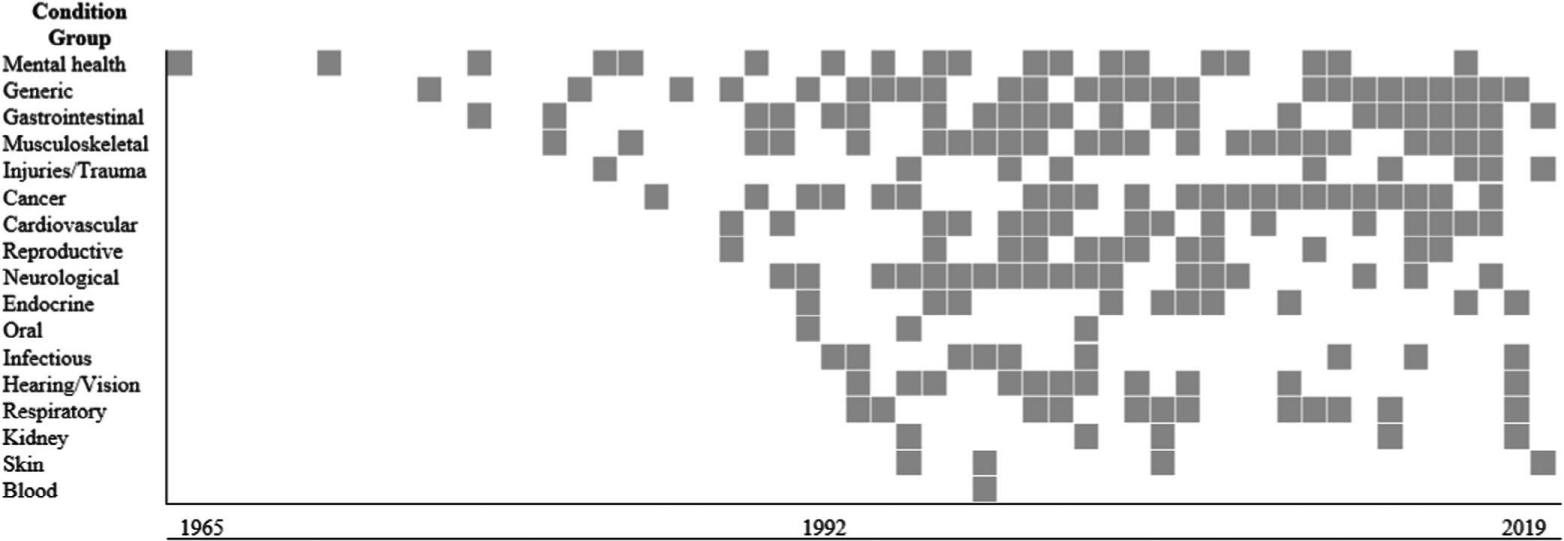
Validation papers by year for each condition group. Shaded grey square represents *at least one* validation paper for a PROM published in that year

Time trends in the publication of PROMs by disease groups are displayed in Figure 4. Among the included conditions, development and validation of PROMs varied by disease group: the first validated mental health PROM was published in 1965, whereas PROMs for other disease groups (eg infectious, hearing and vision, respiratory, kidney, skin and blood) were not developed until the late 1980s and early 1990s.

PROMs meeting our inclusion criteria were originally developed in a range of countries including Singapore, Norway, New Zealand and the Netherlands, although almost half had been developed in the United States of America (n = 147, 46.8%), followed by the United Kingdom (n = 81, 25.8%), Canada (n = 29, 9.2%) and Australia (n = 12, 3.8%). Thirty‐three (10.5%) PROMs had been simultaneously validated in multiple countries and often numerous languages, such as the KIDSCREEN‐52, a generic PROM assessing health‐related quality of life in children and adolescents, validated in 12 European countries (Austria, Czech Republic, France, Germany, Greece, Hungary, the Netherlands, Poland, Spain, Sweden, Switzerland, the United Kingdom)^28^ and the InFLUenza patient‐reported outcome (FLU‐PRO), which assesses influenza symptoms and was validated in English and Spanish across the United States, United Kingdom, Mexico and Argentina.^29^

We also examined the number of citations received by each of the PROM validation papers as an indication of the use of the measure (averaged by year). Overall, validation papers received an average of 43.7 cites per year, but there was a high degree of skewedness (min = 0, max = 1102.4, mdn = 11.3, SD = 108.1). Table 3 displays the number of citations per year for PROM validation papers by disease group. Validation papers from mental health PROMs had the highest number of citations per year at 254.1, followed by generic PROMs (54.7 citations) and blood and metabolic PROMs (47.0 citations), of which there were only two.

**TABLE 3:** Number of cites per year received by each of the PROM validation papers in each condition group, averaged by both year and number of PROMs in that group

Condition groups	Average cites per year
Mental health	254.1
Generic PROM	54.7
Blood and metabolic	47.0
Cancer	39.0
Oral	36.2
Respiratory	34.7
Skin	30.6
Kidney and urinary	29.4
Musculoskeletal	28.4
Neurological	24.8
Reproductive and maternal	24.5
Cardiovascular	16.7
Hearing and vision	15.0
Gastrointestinal	14.2
Endocrine	11.5
Infectious	8.7
Injuries and trauma	7.7

For 51 (16.2%) PROMs, published reports were able to be found online, indicating the use of these PROMs for clinical or policy purposes. Two‐hundred and seventy (85.7%) of the included PROMs had been used in subsequent research studies. This left 45 PROMs with no evidence online to indicate subsequent use following validation, although 21 of these had been published in the last 5 years.

## 4 DISCUSSION

This review identified both generic and condition‐specific PROMs, evaluated trends in measures and publications and considered contemporary issues in the development, validation and application of PROMs in health care. We identified 315 validated PROMs (Appendix S1), covering a range of common conditions with high burden of disease across all major condition groups.^25^ Thirty‐nine of the included measures were generic PROMs.

### 4.1 Trends

There was growth in the academic literature on PROMs, particularly over the last decade, reflecting the broader movement to put the patient at the centre of health care. PROMs value patients as experts on their experiences and can facilitate their involvement in clinical decision making.^2^ The increase in publications on PROMs reflects a growing global recognition that incorporating the patient's perspective is integral to the quality and effectiveness of health care.^30^

The average number of citations received per year for validation papers suggests a strong appetite for using PROMs. Mental health PROMs had the highest average citations per year. This may be because of the centrality of self‐report in diagnosing and monitoring these conditions, which typically lack biological markers; hence, some of the mental health PROMs are also used for screening and diagnosing these conditions.^31^, ^32^, ^33^ Indeed, we found numerous mental health PROMs validated as early as the 1960s, long before PROMs for physical disorders, or before the nomenclature for these measures became established.

Citation counts are just one indicator of the use of a PROM and not one that demonstrates use in clinical practice. We found evidence in reports published online that some of the PROMs reviewed were used in clinical practice or to inform policy. For example, PROMs had been used to evaluate the effectiveness of care delivery models,^34^ understand the needs of particular populations,^35^, ^36^ examine care pathways^37^ and assess treatment effectiveness..^38^, ^39^ As part of the uptake of PROMs in practice, ICHOM, established in 2012, publishes standard sets of outcomes (including patient reported) for different medical conditions, together with implementation advice, that hospitals internationally can use to inform what they measure.

In scoping the literature, we also observed utilization of CAT over the last decade. CAT is able to differentiate respondents along the continuum of a trait (eg degree of pain) by extensive collection, content validation and calibration of item banks.^40^ Calibrating item banks involves advanced psychometrics using item‐response theory to determine the degree of ‘difficulty’ or level of the underlying trait being measured (eg ‘moderate’ pain) for each item.^41^ Using calibrated item banks in computer administered PROMs is thought to improve efficiency, making delivery of the questionnaire more dynamic and flexible because new questions are adapted to patients’ prior responses.^42^ In that sense, CAT‐enabled PROMs are more individualized than traditional PROMs, which have been criticized as having limited flexibility,^43^ being potentially inappropriate for use among some groups,^44^ and lacking timeliness in generating prompt, clinically meaningful interpretations.^45^ The Patient‐Reported Outcome Measure Information System (PROMIS), a National Institute of Health initiative ^46^, ^47^ was the most notable example identified of CAT‐enabled PROMs. PROMIS item banks have been progressively created for adult and paediatric populations across physical (eg fatigue), mental (eg anxiety) and social health (eg peer relationships). In many cases, these were drawn from other validated PROMs, but their calibration, coupled with the extensive support available and limited restrictions on use, has contributed to PROMIS’ popularity.^48^

### 4.2 Issues

Many of the PROMs identified by this review were well‐established—some were more than 30 years old—while papers captured from our search of academic databases were more recent. Thus, the term ‘patient‐reported outcome’ appears to be a fairly new designation for measures, questionnaires and inventories that might once have been described as assessing symptom severity or health‐related quality‐of‐life. It was often only in retrospect, by the attribution of other authors, that older instruments were recognized as PROMs. This may make searching for PROMs challenging.

There were differing degrees of validation and notions of what constituted validity in the PROMs reviewed. Not all forms of validity testing (eg face, content, construct, criterion‐related, known group differences) are sufficient to claim that a scale is valid. There is a growing trend to assess the methodological quality of PROM studies using the COnsensus‐based Standards for the selection of health status Measurement INstruments (COSMIN).^49^, ^50^ Increasingly systematic reviews are being conducted on PROMs for specific conditions and symptoms, and these use COSMIN or other assessment criteria to make determinations about which PROM(s) is superior in terms of validity.^51^, ^52^, ^53^, ^54^ Although it was not the purpose of this review to make such recommendations, it is clear that COSMIN is an increasingly important tool for assessing potential PROMs.

Many PROMs reviewed were not originally developed for use in clinical practice or to inform policy decisions and may have issues with responsiveness to change or potential floor and ceiling effects that limit their potential in these settings.^55^ Recently, there have been calls to reconceptualize the validation of PROMs as an on‐going, iterative process of evidence accumulation, with consideration of how the data will be used to make decisions about individuals and populations, instead of a static designation that a PROM *is* valid.^56^ The findings of our review must be considered in light of this idea; although we identified PROMs validated in English and for the measurement of outcomes for particular conditions, within these parameters there are a broad range of contexts (eg different countries, care settings), populations (eg older patients, condition subtypes) and purposes at the micro‐, meso‐ and macro‐level (eg decision making with individual patients, provider comparison, funding health‐care organizations) for which validity could conceivably vary.

### 4.3 Things to consider when selecting a PROM

As others have noted, a key challenge for researchers, policymakers and clinicians is selecting a reliable and valid tool from the hundreds of PROMs available.^57^ These measures are designed to be subjective and reflect patients' perspectives and experiences; they do not replace other more objective measurements. Rather, PROMs should be used to complement clinical data.^30^

One decision is whether to use a generic or condition‐specific PROM. Limitations exist for both. Generic PROMs can be used to generalize or compare across different conditions but lack sensitivity to condition‐specific outcomes; hence, they may have greater applicability at an organizational or system level. Condition‐specific PROMs are argued to have greater face validity, credibility and responsiveness to changes in the patient's condition.^2^ Hence, they are most appropriate for measuring treatment outcomes within specific clinical populations and focusing on the individual level.^11^ For these reasons, it has been recommended that both types of PROMs be used concurrently and applied at different levels of the health system.^30^ Our review identified nuances and different focuses of outcome domains within condition‐specific PROMs for the same condition. For example, some PROMs for rheumatoid arthritis focus on quality of life (eg^58^), while others prioritize subjective assessments of symptom impacts (eg^59^) or functional status (eg^60^), and some focus on specific symptoms (eg fatigue^61^) or social issues related to the condition (eg work instability^62^). With these issues in mind, selecting the right PROM(s) requires careful reflection on the intended purposes, including why patients’ responses are being collected, what one expects or wants to find, what information is relevant to capture, and how this this information will be used.^63^

### 4.4 Strengths and limitations

The method reported here is a multipronged approach to reviewing the literature and identifying PROMs. A key strength was examining both generic and condition‐specific PROMs to consider trends and issues in this growing field. Less than two thirds of instruments evaluated came from the academic databases search, likely because this search used keywords exclusively related to PROMs rather than, for example, health‐related quality‐of‐life, symptom severity or other more specific terms that are now captured under the umbrella of ‘PROMs’. This weakness was to some extent mitigated by additional searches including snowballing, scoping published reviews and searching grey literature. However, some potential instruments meeting our criterion (1) of assessing health outcomes reported directly by patients may have been missed by our search strategy if they are no longer used or have never been recognized as PROMs.

This review synthesized trends across 315 PROMs, including both generic and condition‐specific, but due to the rapid nature of the review, the collection of some information, particularly the use of a PROM in published reports or exemplary studies, was not exhaustive, nor is this aspect of the process entirely replicable. These insights should be treated as indicative of how PROMs are being used generally, rather than a clear demonstration of *which* PROMs are used in research and practice and which are not. New PROMs continue to be published, including for new conditions and clinical areas.^64^ We excluded a number of measures that are still in the early stages of validation (eg reporting on content validity) but it is likely that over time, through more extensive validation, these would have met criteria for inclusion. We also excluded PROMs where we could not find evidence of English validation, even though some had translations available.^65^ The research on, and use of, PROMs, as this review has shown, is constantly moving.

## 5 CONCLUSION

There are many PROMs and many more studies examining, using or discussing them. In this review, we identified 315 generic and condition‐specific PROMs, producing a library available for public use that will be updated over time.^66^ We also evaluated trends among measures and publications on PROMs and discussed contemporary issues related to their development, validation and application in healthcare. A key challenge to using PROMs is selecting a reliable and valid tool that is appropriate for one's purpose from the hundreds of instruments available. This review provides insights to assist with understanding the scope of available generic and selected condition‐specific PROMs, including trends in the development, validation and use of these PROMs. It highlights the growing global recognition that incorporating the patient's perspective is integral to the quality and effectiveness of health care and issues associated with this shift. The review demonstrates that the measurement of patient‐reported outcomes is an evolving field.

## CONFLICT OF INTERESTS

None declared.

## AUTHOR CONTRIBUTIONS

SH, LM and CL conceptualized the project. KC, CP and LE conducted the literature review and drafted the manuscript with support from SH and LM. KC, CP, LE, JL, SH, LM, CL and JB contributed to the final version of the manuscript.

## Supporting information

Appendix S1

## Data Availability

The data that support the findings of this study are available in the supplementary material Appendix S1 of this article.
